# Ongoing debate on data governance principles for achieving Universal Health Coverage: a proposal to post-G20 Osaka Summit meetings

**DOI:** 10.1080/16549716.2020.1859822

**Published:** 2020-12-18

**Authors:** Shuhei Nomura, Haruka Sakamoto, Aya Ishizuka, Yasushi Katsuma, Hidechika Akashi, Hiroaki Miyata

**Affiliations:** a Department of Health Policy and Management, School of Medicine, Keio University, Tokyo, Japan; b Department of Global Health Policy, Graduate School of Medicine, The University of Tokyo, Tokyo, Japan; c Research Center for Community Health, Minamisoma Municipal General Hospital, Fukushima, Japan; d Institute for Global Health Policy Research (iGHP), National Center for Global Health and Medicine, Tokyo, Japan; e School of Global Studies and Collaboration, Aoyama Gakuin University, Kanagawa, Japan; f Graduate School of Asia-Pacific Studies, Waseda University, Tokyo, Japan; g Bureau of International Health Cooperation, National Center for Global Health and Medicine, Tokyo, Japan; h Department of Healthcare Quality Assessment, Graduate School of Medicine, The University of Tokyo, Tokyo, Japan

**Keywords:** Data governance, data health, universal health coverage, Osaka Track, Data Free Flow with Trust

## Abstract

The Group of 20 Summit (G20) in Osaka, which Japan chaired for the first time in June 2019 has created a tailwind for achieving universal health coverage (UHC) globally. In response to the rapid digitalization, the G20 leaders commenced negotiations for the Osaka Track framework to formulate international rules on data flow across borders and systematize the concept of ‘Data Free Flow with Trust (DFFT).’ The strategic harnessing of the power of data to strengthen the healthcare system can allow for rapid and affordable progress toward achieving UHC. However, world leaders have yet to discuss what data governance approaches the Osaka Track will follow, or even on what values it will seek to create and maximize. In this paper, we propose a people-centered, trust-oriented approach as the key principle of data governance toward achieving UHC, using Japan’s experience as an example. We believe that this approach is compatible with other prevailing approaches (e.g. the General Data Protection Regulation (GDPR) in the European Union), and can serve as a bridge to their conceptual differences. We hope that our proposed principles will be fully discussed in post-G20 Osaka Summit meetings.

## Beginning of the era of global data governance

Achieving universal health coverage (UHC), namely where all people have equitable access to quality health services without financial hardship, is positioned as the Target 3.8 in the Sustainable Development Goals (SDGs) set forth by the United Nations (UN) [1]. In 2019, stakeholders experienced a historical momentum for UHC. At the UN High-Level Meeting on UHC, member states agreed upon a political declaration that emphasized the importance of utilizing data to achieve evidence-based decisions and policies on health, while also addressing the need for protecting data and privacy and narrowing the digital divide [2]. In 2018, the UN Secretary-General had already highlighted the importance of transparency and inclusion in his ‘Strategy on New Technologies’ [3], deeming these concepts as part of the five key principles for the UN to better harness technology and innovation in the quest for achieving SDGs.

In the same year of 2019, in Osaka, The Group of 20 (G20) Summit provided another impetus for achieving UHC. G20 is an international forum for the governments and central bank governors from 19 countries and the European Union (EU), and G20 Summits are held once a year to discuss a diverse range of topics relevant to each G20 member country, including global health. Originally, the G20 Summit was held for advanced and emerging economies to discuss and respond to the global financial crisis that occurred in 2008; since then, it has expanded its scope to cover other global challenges. In response to the rapid digitalization of societies worldwide, the member countries announced, at the 2019 G20 Summit, the onset of negotiations that would begin to establish international rules for the free flow of data across borders. This track follows the Data Free Flow with Trust (DFFT) notion, which dictates that trust is a driving factor that enables the free flow of data. It was first espoused by Japanese former Prime Minister Shinzo Abe in his speech at the World Economic Forum’s annual meeting in Davos, Switzerland in January 2019 [4]. As indicated in the recent World Health Organization (WHO)’s guideline on the use of digital health technologies entitled ‘WHO Guideline: recommendations on digital interventions for health system strengthening,’ a strategic harnessing of the power of data to strengthen healthcare systems can make for rapid and affordable progress towards achieving UHC [5].

The year 2019, thus, not only marked the beginning of global data governance, as stated in Shinzo Abe’s speech in Davos, but also was undoubtedly an important year for the advancement of UHC. Following the momentum, many countries are currently and increasingly turning to data to help guide the development and strengthening of UHC. The Political Declaration of the High-Level Meeting on UHC also emphasized that strengthening health information systems and collecting good quality data are key actions to monitor the progress and identify the gaps in the universal and inclusive achievement of SDGs, especially those related to health SDGs. Quality and sufficient data has been shown to enable governments to properly monitor their disease management, financial management, and claims data utilization, all of which contribute toward UHC [6]. For example, in 2011, Kenya launched Africa’s first open data portal [7], and other African countries have since been following this movement [8]. However, many countries, especially low- and middle-income countries (LMICs), still face complicated, foundational challenges to harness the value of their health data. One of the underlying causes of such challenges is the lack of a widely accepted concept for data governance, especially on the most appropriate person/institution to have control over individual data and on the underlying values that should serve as framework for data governance. As shown by Nicki et al.’s on the four key domains for better data governance in LMICs, the major topics that should be addressed are the assurance of data protection through data access controls and application of the relevant legislation [9]. In this paper, we used the case of Japan – as an example – to propose key principles that may serve to guide data governance for achieving UHC.

## A paradigm shift in data governance

The retention and utilization of personal data by select entities, even when individuals give consent for the processing of their personal data, can create a power imbalance between the data holding entities and those without data access; this power covers a diverse range of dimensions, including economic and political powers. Such power imbalance often raises issues pertaining to data protection, transparency, and accountability, and can hamper the development of innovative forms of data usage for research, business, journalism, etc. Today, several principles on governance for personal data already exist – the principal approaches of data governance and their value focuses and challenges are shown in Table 1 [10–12]. In this study, we used the following four data governance approaches as examples: privacy-, economy-, social value-, and people-/trust-centered approaches. The privacy- and economy-centered approaches, which are adopted by the EU and the USA (US), have been widely used globally. We also chose to use China’s social value-centered approach as an example because it is based on concept that is largely different from those found in Western societies. Finally, we explored Japan’s people-/trust-centered approach because it is different from the former three.

**Table 1.** Principal data governance approaches and their value focuses and challenges

	European Union	USA	China	Japan
Approach	Privacy-centered	Economy-centered	Social value-centered	People-/Trust-centered
Value	The right of individuals to control their data (e.g. General Data Protection Regulation: GDPR)	Innovation through rational corporate activities	Visualization and sharing of social values	People centered, and trust oriented
Challenge	Exclusive ownership of personal data	Data hegemony	Top-down and unified social value	

The privacy-centered approach to data governance is embodied by the General Data Protection Regulation (GDPR), which was enacted in the EU in May 2018 [13]. GDPR covers the handling of personal data and guarantees the rights of all citizens in the EU to control the range and usage of the personal data that they provide. At its core, we can observe the concepts of data portability and access rights, which are important elements that could become fundamental human rights in the twenty-first century. While the GDPR recognizes the value of data as a common and public good, it also provides a strong underlying notion that personal data is an exclusive property of an individual. Individuals’ strong control over data, however, may hinder the efficient use of data in the market [9,10]. Despite its strengths (e.g. thorough protection of personal information and consideration of privacy), the GDPR also presents controversies, especially on harmonization among different sectors [14]. For example, although the GDPR inhibits the handling of personal data in some cases, it allows for the processing of the same data in others, thereby detracting its harmonization. Moreover, the GDPR has other concerns, such as the following: complicated processes that are difficult to be applied to cross-border healthcare challenges; technical difficulties that hinder researchers’ ability to obtain informed consent for clinical trials and research according to GDPR standards; and unclear evidence on the transferability of data under GDPR [14,15].

The economy-centered approach to data governance is espoused by the US, emphasizing the creation of market-driven value of the business [10,11]. The social value-centered approach, on the other hand, aims to utilize personal data to build trust and accountability in society, and it is exemplified by the Social Credit System in China [11,12]. Despite these varying approaches and as nations worldwide move toward a more surveillance-oriented society, world leaders need to start tackling the common challenges that are shared by these data governance approaches; for example, the possibility of data hegemony by giant technological corporations, and the risk of diluted diversity of individual values and beliefs.

## The proposal – a person-centered, trust-oriented approach

Currently, major international conferences or dialogues among high-level government officials have yet to address what types of data governance approaches that the Osaka Track and DFFT will follow (or create on their own), or on what values they will seek to create and focus. Thus, by building upon the Osaka Declaration on Digital Economy and the delineations presented at the G20 Trade Ministers and Digital Economy Ministers meeting (which occurred in June 2019), we propose a data governance approach for achieving UHC that is both people-centered and trust-oriented; namely, data should be integrated at the individual level (i.e. people should have the ownership over their own health data) and any public/private organizations should be enable to use the individuals’ data only upon the obtainment of individual informed consent. We believe this approach to be compatible with the value focuses of the other three prior approaches (i.e. used by the EU, US, and China), and one that can bridge their conceptual differences.

A people-centered approach translates to a data governance that highly regards individual values. In healthcare, the people-centered approach empowers people to actively participate in and design their own health. For example, if consent is given, individual-level data held by different entities (e.g. insurers and hospitals) can be integrated and applied for the delivery of a more personalized healthcare. A wider use of healthcare data integrated around individuals can also improve access to healthcare and the effectiveness of the service coverage, resulting in a reduced healthcare cost [5]. These are the characteristics of the three dimensions of the UHC (i.e. population covered, services covered and its quality, and costs covered). Meanwhile, it should be acknowledged that there are transformational challenges brought about by a data-driven society, particularly in the LMICs; exemplifying, limited data infrastructure, literacy, and expensive access to data processing tools and software, etc. [16].

A trust-oriented approach means that trust must be secured in the data flow between people, communities, businesses, governments, and/or other social networks. Trust is developed, fostered, and maintained through the rigorous protection of personal data, which can, in turn, be guaranteed by law or policy. Currently, laws and policies are not always in place to ensure that data governance, especially in the healthcare sector, is applied in a way that adequately protects personal data [17]. As exemplified by the 2018 Facebook’s Cambridge Analytica scandal, people will steer away from services if their privacy and trust are being breached [18]. Trust is, therefore, an important resource in a data-driven society; this is most special in the LMICs, where privacy and data protection rules have not yet been established or enforced by law. This approach reflects the notion of DFFT proposed by Shinzo Abe [4].

## A case study of Japan

In Japan, the characteristics of the healthcare system can be summarized under the following concepts: a universal insurance system (i.e. those who are living in Japan are obliged to enroll in the universal insurance system), an uniform fee schedule (i.e. the central government determines the price of healthcare process, which is applicable to both public and private healthcare facilities), and a lack of a strict gatekeeping system [19]. In 1961, the Japanese government introduced this universal health insurance system. In it, people living in Japan are required by law to enroll in the universal health insurance and pay a 0–30% co-payment whenever they seek healthcare services. Moreover, private health insurances only play a supplementary role, such as to cover the co-payment for hospitalization fees and the transportation fees for hospital visits.

As for the service fees, the government sets the prices of healthcare procedures and medicines covered under the universal insurance system; this specific fee is called the ‘uniform fee schedule.’ This uniform fee schedule is applicable not only for public but also for private institutions (i.e. individual providers, even for private healthcare facilities, cannot negotiate the contractual terms set forth by the government under the universal health insurance). Through this methodology, Japan has provided equitable access to healthcare to its population at an affordable cost, and successfully controlled the total health expenditure with the uniform fee schedule.

Another characteristic of the Japanese healthcare system is that it does not have a ‘gatekeeping system,’ which most European countries have; instead, people in Japan can freely choose to which healthcare facilities they want to go. This means that, even if people are experiencing a mild to a moderate symptom, they can go to university hospitals or other tertiary healthcare facilities. Additionally, other forms of insurance – namely, any other forms that could substitute the universal health insurance – have been banned in Japan; as remarked, private health insurance companies only play a supplementary role (e.g. covering the co-payment for hospitalization fees, the transportation fee for hospital visits, or the wages lost during the days that the person was hospitalized).

Nonetheless, half a century after the achievement of UHC in Japan through the provision of the universal health insurance, the sustainability of the national healthcare system is now being threatened by population aging, escalating health expenditures, etc. [20]. To re-shape the healthcare system and make it sustainable, in 2015, a healthcare advisory panel to the Minister of Health, Labour and Welfare (MHLW) created the ‘Health Care 2035,’ which delineated the future of the healthcare system in Japan; in it, the power of data was positioned as an important pillar [21]. The new national growth strategy, called ‘Future Investment Strategy 2018,’ also requires a change to a data-driven ‘Society 5.0’ (which is interlocked with the SDGs) that utilizes big data and new technologies (i.e. developed through the information and communications technology [ICT]) through a people-centered, trust-oriented approach. As one of the flagship projects leading this change, the government proposed the development of a new healthcare system that is fully data-driven [22].

For example, a Person-centred Open PLatform for wellbeing (PeOPLe) – a platform proposed by an ICT advisory panel to the MHLW in 2016; a panel which included HM, the author of this paper – is planned to be introduced in 2020 in a stepwise manner [23]. PeOPLe is a nation-wide platform that aims to enable the public to openly utilize all kinds of data. The proposal was published as a government report on the website of the MHLW [23]. Through it, the data is supposed to be managed in a distributed fashion and anyone will be able to connect to it on an individual basis using a unique individual citizen identifier (i.e. person-id) in the platform thus de-monopolizing the government and its power over people’s data. In 2019, the government decided that the already existing citizen identifier (i.e. ‘My Number’) in Japan, which has been used in the social security and tax system that started in 2016 would be implemented as the person-id in the PeOPLe. Through the PeOPle, individuals will have access to various public data, such as public health check-ups, health insurance claims, and long-term care insurance claims. The platform will also connect people’s personal health records and, in the future, various life-logs and sensing data that are collected through the enterprise Internet of Things (IoT).

By gathering all individual’s data and by giving them the control over their own health data, the government expects that the individuals can become more aware about their health status, thereby allowing them to utilize the data to positively change their health behaviors. Additionally, given that the government (i.e. from the central to local level) is also allowed to access the data, they can utilize this data for various endeavors related to health policy planning (e.g. for managing health financing at the regional and local level). Notwithstanding, although the data is gathered at the individual level, there is an ongoing debate for a system that allows the government, healthcare facilities, and other relevant organizations to utilize the data without any consent from the individuals under specific circumstances; specifically, this could prove useful in cases of natural disasters and other emergencies – in which data usage may directly contribute to saving lives.

Still, the rules for operational methods and data utilization remain under development. Once the PeOPLe is established, Japanese individuals may be able to grant access to their health data (in an anonymized form) by opting-in, or -out from research. Moreover, healthcare professionals may be able to access individual’s personal health records up to the level of disclosure. Summarizing, the PeOPLe is expected to ensure equitable access to data, effectively promote personalized healthcare and social care, and help stakeholders address the social determinants of health that go beyond traditional disease management measures. Additionally, it has the potential to reduce costs through resource usage and through allowing for the deployment of on-demand care services, all of which directly reflect the goals of UHC.

The PeOPLe will incorporate Japan’s data protection laws in its general business terms, thus specifying the privilege of data use according to the nature of users’ objectives. Additionally, by adhering to the national laws on data protection, any type of personal identification information (e.g. person-id) will automatically be detracted from data prior to being accessed, thereby offering users a perfectly anonymized dataset.

Previous studies support the potential health impact of the PeOPLe. For example, there is evidence that good follow-up of patients’ postoperative prognosis leads to prolonged survival [24]. Furthermore, decision aids based on a people-centered approach were shown to evoke the following: improve patients’ understanding of treatment options minimize conflicts arising from lack of information or unclear value sets [25]; help encourage patients’ active participation in treatment decisions; and effectively help patients to appropriately recognize treatment-related health risks [25]. Although few empirical studies have been conducted, one study showed that people-centered information-sharing through ICT may be effective in maintaining and improving patients’ health [26]; another study reported increased patient empowerment and health-related quality of life after patients used people-centered information sharing through ICT [27].

## Application to LMICs for attaining UHC

As indicated in several milestone documents about UHC (e.g. the UN High Level Political Declaration on UHC), utilizing health data is key for successful UHC; accordingly many countries are making efforts to attain better data governance to ensure proper use of such health data. Still, several countries across the globe, especially LMICs, are currently facing several challenges related to data governance; these range from understanding and formulating optimal policies/legislations to determining the underlying core values behind these policies/legislations. Concomitantly, high-income countries have recently begun to develop various laws on data governance, something done generally ahead of LMICs, so many lessons can be extracted, learned, and applied from these progresses [9,28]. Overall, when endeavoring to develop a UHC, the first step is to determine its core values; the second is to define who owns the data (i.e. whether it is owned by individuals, the government, or private companies); and the third is to determine who can use the accumulated data, to what extent the data can be used, and what would be the mechanisms of access.

As the Japanese example shows, in principle, the people-centered, and trust-oriented approaches can be core values of the UHC, aiming to ensure individual ownership of data. On one hand, this allows for the empowerment of the Japanese population, who can then sense and experience greater control over their own health and wellbeing. As represented above, this approach differs from that in the US (i.e. where private companies own the data) and from that in China (i.e. where the government owns the data). On the other hand, to avoid overly strong personal protections that could prevent the use of data for innovation and wellbeing improvements, the Japanese system concomitantly allows for some institutions (e.g. public institutions, universities, private companies, etc.) to use the data, even if under the condition of individuals giving their informed consent for such use. This system is based on trust among the involved parties.

If LMICs endeavor to adopt a system that strives for a balance between personal data protection and data utilization, we believe that the Japanese system, which is based on trust, may serve as an useful example. The underlying values that will guide the data governance of each country will largely depend on each countries’ individual cultural and social backgrounds; thus, we acknowledge that applying the Japanese values directly to another country is not always possible to optimal. However, we hope that the Japanese values can serve as a framework for introducing a new set of values that differs from the mainstream ones in the EU, the US, and China, and namely can be used as another point of reference. Accordingly, future studies are warranted to examine the values tailored specific to each LMIC should be proposed and tested for their usefulness.

## Conclusion

In summary, we proposed a data governance that is focused on a people-centered and trust-oriented approach, which may allow for achieving UHC in the era of a data-driven society. Many countries, especially LMICs, are endeavoring toward UHC, which requires the development of data governance that prove more efficient and secure. Given that the key for success on improved data governance relies on its underlying core values, we proposed Japan’s balanced approach – which is based on a principle of trust – as one option for these countries. We chose this specific approach because we believe that the principle of trust resonates with the notion of UHC and with the SDGs. We believe that, if the G20 Summits and other high-level meetings attempt to clarify the principles that are required promote a coordinated action on data governance and UHC, it may possible to promote cooperation at the national, regional, and global levels. We hope that these proposed principles will be fully discussed in post-G20 Osaka Summit meetings.

## Paper context

The world has turned into a data-driven society, which holds significant implications for the healthcare sector. In this paper, we summarize key principles behind data governance practiced in China, EU, USA, and Japan; and propose a ’people-centered, trust-oriented approach’ as a critical data governance principle in the area of healthcare. This approach reflects the Sustainable Development Goal’s notion of leaving no one behind and will actively promote the achievement of universal health coverage.

## References

[1] United Nation. Sustainable development, the 17 goals. New York: United Nation. Available from: https://sdgs.un.org/goals

[2] United Nation. Political declaration of the high-level meeting on Universal Health Coverage. New York: United Nation; 2019. Available from: https://www.un.org/pga/73/wp-content/uploads/sites/53/2019/07/FINAL-draft-UHC-Political-Declaration.pdf

[3] United Nation. UN secretary general’s strategy on new technologies. New York: United Nations; 2018. Available from: https://www.un.org/en/newtechnologies/images/pdf/SGs-Strategy-on-New-Technologies.pdf

[4] World Economic Forum. ‘Defeatism about Japan is now defeated’: read Abe’s Davos speech in full. World Economic Forum; 2019. Available from: https://www.weforum.org/agenda/2019/01/abe-speech-transcript/

[5] World Health Organization. WHO guideline: recommendations on digital interventions for health system strengthening. Geneva: World Health Organization; 2019. 31162915

[6] Joint Learning Network for Universal Health Coverage. Using health data to improve universal health coverage three case studies. Virginia: Joint Learning Network for Universal Health Coverage; 2018.

[7] The World Bank. Government of Kenya releases data to public on easy to use web portal. Washington, DC: The World Bank; 2011. Available from: https://www.worldbank.org/en/news/press-release/2011/07/08/government-kenya-releases-data-public-easy-use-web-portal

[8] World Wide Web Foundation. Open data barometer - leaders edition. Washington, DC: World Wide Web Foundation; 2018.

[9] Tiffin N, George A, LeFevre AE. How to use relevant data for maximal benefit with minimal risk: digital health data governance to protect vulnerable populations in low-income and middle-income countries. BMJ Glob Health. 2019;4(2):e001395. 10.1136/bmjgh-2019-001395 PMC6509603 31139457

[10] Tankard C. What the GDPR means for businesses. Network Secur. 2016;2016(6):5–6.

[11] Chen L, Cheng W, Ciuriak D, et al. The Digital Economy for Economic Development: Free Flow of Data and Supporting Policies. Task Force 8: Trade, Investment and Globalization; 2019. Available from: https://t20japan.org/wp-content/uploads/2019/03/t20-japan-tf8-4-digital-economy-economic-development.pdf.

[12] Kobie N. The complicated truth about China’s social credit system. WIRED; 2019. Available from: https://www.wired.co.uk/article/china-social-credit-system-explained

[13] Haug CJ. Turning the tables - the New European general data protection regulation. N Engl J Med. 2018;379(3):207–209. 29874143 10.1056/NEJMp1806637

[14] Chico V. The impact of the general data protection regulation on health research. Br Med Bull. 2018;128(1):109–118. 30445448 10.1093/bmb/ldy038

[15] Prata Ribeiro H, Ponte A, Robalo Cordeiro F, et al. [The new general data protection regulation and its implications regarding clinical information requests to healthcare professionals]. Acta Med Port. 2020;33(4):221–224. 32238234 10.20344/amp.13162

[16] Bloom G, Berdou E, Standing H, et al. ICTs and the challenge of health system transition in low and middle-income countries. Global Health. 2017;13(1):56. 28784144 10.1186/s12992-017-0276-y PMC5547477

[17] Jain P, Gyanchandani M, Khare N. Big data privacy: a technological perspective and review. J Big Data. 2016;3(1):25.

[18] The Guardian. Revealed: 50 million Facebook profiles harvested for Cambridge Analytica in major data breach: the Guardian. 2018. Available from: https://www.theguardian.com/news/2018/mar/17/cambridge-analytica-facebook-influence-us-election

[19] Sakamoto H, Rahman M, Nomura S, et al. Japan health system review. New Delhi: World Health Organization, Regional Office for South-East Asia; 2018.

[20] Japan: universal health care at 50 years. The Lancet. Japan: universal health care at 50 years. Lancet. 2011;378(9796):1049. 10.1016/S0140-6736(11)61223-3 21885103

[21] Miyata H, Ezoe S, Hori M, et al. Japan’s vision for health care in 2035. Lancet. 2015;385(9987):2549–2550. 26122147 10.1016/S0140-6736(15)61135-7

[22] Prime Minister of Japan and his Cabinet. Future investment strategy 2018. Tokyo: Prime Minister of Japan and his Cabinet; 2018. Available from: https://www.kantei.go.jp/jp/singi/keizaisaisei/pdf/miraitousi2018_en.pdf

[23] Ministry of Health, Labour and Welfare. Towards the construction of “next generation health care system” utilizing ICT [Japanese]. Tokyo: Ministry of Health, Labour and Welfare; 2016. Available from: https://www.mhlw.go.jp/file/05-Shingikai-12601000-Seisakutoukatsukan-Sanjikanshitsu_Shakaihoshoutantou/0000140306.pdf

[24] Basch E, Deal AM, Dueck AC, et al. Overall survival results of a trial assessing patient-reported outcomes for symptom monitoring during routine cancer treatment. JAMA. 2017;318(2):197–198. 28586821 10.1001/jama.2017.7156 PMC5817466

[25] Stacey D, Legare F, Lewis K, et al. Decision aids for people facing health treatment or screening decisions. Cochrane Database Syst Rev. 2017;4:CD001431. 28402085 10.1002/14651858.CD001431.pub5 PMC6478132

[26] Walsh S, Golden E, Priebe S. Systematic review of patients’ participation in and experiences of technology-based monitoring of mental health symptoms in the community. BMJ Open. 2016;6(6):e008362. 10.1136/bmjopen-2015-008362 PMC4916567 27329437

[27] Wildevuur SE, Simonse LW. Information and communication technology-enabled person-centered care for the “big five” chronic conditions: scoping review. J Med Internet Res. 2015;17(3):e77. 25831199 10.2196/jmir.3687 PMC4393506

[28] Fernando B, King M, Sumathipala A. Advancing good governance in data sharing and biobanking - international aspects. Wellcome Open Res. 2019;4:184. 31950088 10.12688/wellcomeopenres.15540.1 PMC6945104

